# Impact of rehabilitation trajectory on affective and cognitive impairment after intracerebral hemorrhage: a cohort study

**DOI:** 10.3389/fneur.2026.1829901

**Published:** 2026-06-10

**Authors:** Qiuyi Jiang, Guangyao Shi, Hongli Zhang, Shouyue Wu, Chunyang Liu, Jian Zhang, Enzhou Lu, Chao Yuan, Yanchao Liang, Lu Wang, Guang Yang

**Affiliations:** 1 Department of Neurosurgery, The First Affiliated Hospital of Harbin Medical University, Harbin, Heilongjiang, China; 2 Heilongjiang Province Neuroscience Institute, Harbin, Heilongjiang, China; 3 Department of Urology (Heilongjiang Key Laboratory of Scientific Research in Urology), The Fourth Affiliated Hospital of Harbin Medical University, Harbin, Heilongjiang, China

**Keywords:** anxiety symptoms, cognitive impairment, depressive symptoms, intracerebral hemorrhage, neuropsychiatric outcome, rehabilitation trajectory

## Abstract

**Background:**

Spontaneous intracerebral hemorrhage (ICH) significantly affects cognitive and affective functions, with most studies focusing on factors present at or before the index event. This study examines the impact of rehabilitation trajectories within 12 months on cognitive impairment, depression, and anxiety in supratentorial ICH patients with small hematomas.

**Methods:**

This cohort study included 1,692 ICH patients treated between January 2018 and December 2020. Neurological rehabilitation was assessed using the modified Rankin Scale (mRS) at 3, 6, and 12 months, with mRS trajectories identified through a growth-based trajectory model. Affective and cognitive impairments were measured using standardized questionnaires, and their association with different mRS trajectories was analyzed using multivariate logistic regression.

**Results:**

1,563 patients were divided into two trajectories: early rehabilitation (75.5%) and late rehabilitation (24.5%). The late-rehabilitation group showed a higher prevalence of cognitive impairment and depression. After adjustment, late rehabilitation was linked to increased odds of cognitive impairment (OR 1.40, 95% CI 1.02–1.93, *P* = 0.036) and depression (OR 1.96, 95% CI 1.47–2.62, *P* < 0.001), but not anxiety. Subgroup analysis showed that males (OR 1.32, 95% CI 0.95–1.84, *P* = 0.003), hematoma volume ≤10.3 ml (OR 1.91, 95% CI 1.22–3.07, *P* = 0.014), third ventricle Sylvian fissure distance >38.48 mm (OR 2.04, 95% CI 1.36–3.08, *P* = 0.012), and higher education (OR 2.45, 95% CI 1.46–4.19, *P* = 0.016) were more prone to impairments in late rehabilitation group.

**Conclusion:**

Early and sustained rehabilitation is crucial for reducing long-term cognitive and affective impairments following ICH. Personalized strategies are essential, especially for patients on a late-rehabilitation trajectory, to improve neuropsychiatric outcomes.

## Graphical Abstract

**Figure:**
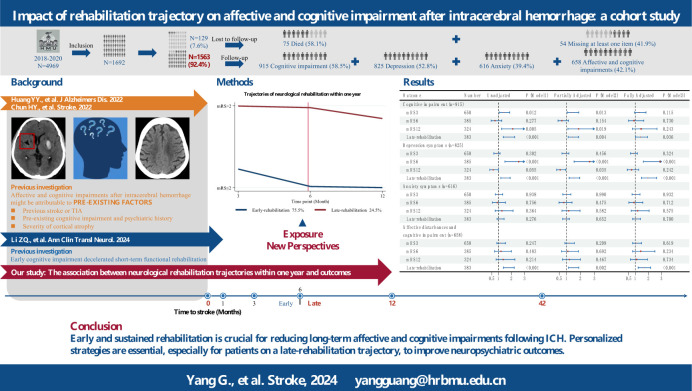
This study examined the impact of neurological rehabilitation trajectories on affective and cognitive impairments in patients with spontaneous supratentorial intracerebral hemorrhage. The results indicate that rehabilitation trajectories over a 12-month period significantly influence long-term neuropsychiatric outcomes, including cognitive impairment, depression, and anxiety. Using Group-Based Trajectory Modeling and Inverse Probability of Treatment Weighting, we found that early and sustained rehabilitation was associated with a lower prevalence of cognitive impairment and anxiety, whereas late rehabilitation was linked to a higher risk of cognitive impairment and depression. These findings highlight the importance of personalized rehabilitation strategies, particularly for patients following a late-rehabilitation trajectory, to improve long-term neuropsychiatric outcomes and reduce the burden of affective and cognitive deficits.

## Introduction

Spontaneous intracerebral hemorrhage (ICH) remains a leading cause of mortality and long-term disability globally (1, 2). While much of the existing research focuses on early functional outcomes, particularly within the first 3 months post-ICH, there is growing evidence suggesting that rehabilitation trajectories can extend well beyond this period, potentially influencing long-term neurological and psychological outcomes (3, 4). Factors such as hematoma location and volume are well-established predictors of poor prognosis, with larger hematomas (>30 ml) and subtentorial hemorrhages associated with worse outcomes (5). Theoretically, selecting patients based on these factors can better guide treatment to maximize benefits (6).

The prevalence of affective and cognitive impairments following ICH is notably high and persistent, significantly impacting patients' quality of life and long-term prognosis (7, 8). These impairments are often categorized into early and late stages, with early impairments typically occurring within the first 6 months post-ICH, and late impairments emerging thereafter (9). Recent research has highlighted that early cognitive impairment is common among ICH patients, significantly slowing short-term functional rehabilitation and serving as an independent predictor of poor outcomes (10).

However, much of the existing research primarily focuses on factors present at or before the index event, often neglecting the impact of neurological rehabilitation on late-stage affective and cognitive impairments (11–13). The interaction between late neurological rehabilitation and these impairments remains underexplored.

This study aims to investigate the impact of varying neurological rehabilitation trajectories within 12 months on the prevalence of cognitive impairment, depression, and anxiety in patients who experienced a supratentorial ICH with small hematomas. Utilizing group-based trajectory modeling (GBTM) and inverse probability of treatment weighting (IPTW) to balance confounding variables, we seek to elucidate the complex interplay between rehabilitation trajectories and neuropsychiatric outcomes. The findings of this study are expected to provide valuable insights for optimizing post-ICH care and identifying patients at high risk for persistent cognitive and affective impairments, thus guiding early interventions to improve long-term outcomes.

## Materials and methods

### Cohort and data collection

**Figure 1:**
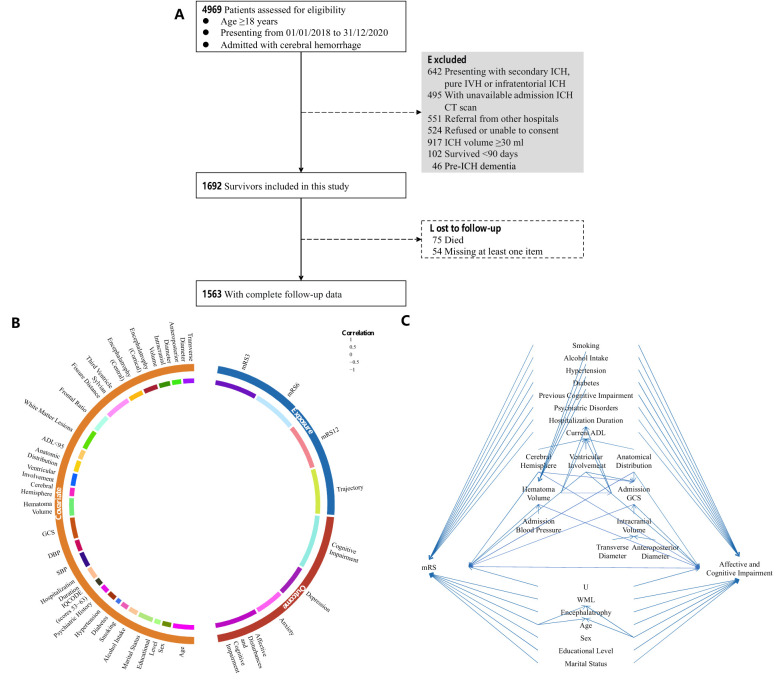
Patient selection flowchart and relationship of study variables. **(A)** Participants flow chart of our study. **(B)** Correlation analysis of covariates with exposure and outcome. **(C)** Directed Acyclic Graph of the association between the modified Rankin Scale and affective and cognitive impairments. CT, computed tomography; ICH, intracerebral hemorrhage; IVH, intraventricular hemorrhage; IQCODE, informant questionnaire on cognitive decline in the elderly; SBP, systolic blood pressure; DBP, diastolic blood pressure; GCS, Glasgow Coma scale; ADL, activities of daily living scale; mRS, the modified Rankin scale.

This observational cohort study included 1,692 adults with ICH treated at the Neurosurgery Department of the First Affiliated Hospital of Harbin Medical University from January 1, 2018, to December 31, 2020, with a mean follow-up period of 3.40±0.93 years. Detailed inclusion and exclusion criteria were shown in Figure 1A.

### Exposure

Neurological functional rehabilitation of stroke patients was assessed at 3, 6, and 12 months after discharge using the modified Rankin Scale (mRS), which ranges from 0 to 6 points. A score of 0 indicates no symptoms at all, while a score of 6 indicates death, with higher scores reflecting worse functional states. The mRS was used as a binary categorical variable, where a value of ≤ 2 indicated favorable outcomes (14).

### Outcome

Structured interviews were conducted by trained neurosurgeons via telephone or video to obtain study outcomes. Cognitive impairment was defined as a Modified Telephone Interview for Cognitive Status (TICS-m) score < 32 (15). Depressive symptoms were defined as a Hamilton Depression Scale (HAMD) score >7 (16). Anxiety symptoms were defined as a Hamilton Anxiety Scale (HAMA) score >7 (16). Affective disturbances and cognitive impairment were identified by the simultaneous presence of cognitive impairment and symptoms of depression or anxiety in patients.

### Covariates

Demographic information and clinical characteristics of the sample were derived from electronic medical records, including age, sex, educational level (lower ≤ 9 years and higher >9 years), marital status (married, divorced/living alone, widowed), personal history (smoking and alcohol intake), medical history (hypertension, diabetes, psychiatric history, and previous cognitive impairment), hospitalization duration, admission blood pressure, admission Glasgow Coma Scale (GCS) score, current activities of daily living (ADL) score, and radiological assessment. Previous cognitive levels were assessed using the short Informant Questionnaire on Cognitive Decline in the Elderly (IQCODE) (17). Scores of 53–63 indicated cognitive impairment, while scores >63 suggested dementia. The ADL evaluated daily function after discharge, with scores < 95 indicating impairment.

### Radiological assessment

Professionally trained neurosurgeons independently evaluated the reformatted head CT images using a standard axial format with the orbitomeatal line as the baseline (18). We obtained the following indices from standardized CT images: hematoma volume, location (cerebral hemisphere, deep ICH, and lobar ICH) (19), ventricular involvement, white matter lesions (WML), encephalatrophy (frontal ratio, third ventricle Sylvian fissure distance, central and cortical brain atrophy), intracranial volume, anteroposterior diameter, and transverse diameter. WML was assessed with the Van Swieten score: 0 indicates no WML, while 1–4 indicates the presence of WML. The severity of brain atrophy was positively correlated with the frontal ratio and negatively correlated with the third ventricle Sylvian fissure distance (20). Anteroposterior and transverse diameter were the maximum distances measurable on CT scans. The total intracranial volume was calculated after 3D Slicer automatically identified and reconstructed the 3D model layer by layer (21).

### Statistical analysis

All statistical analyses were performed using Stata 17.0 (StataCorp LLC, College Station, TX, USA) and R version 4.2.2 (R Foundation for Statistical Computing, Vienna, Austria). Continuous variables were described as mean ± SD or median [interquartile range (IQR)], and comparisons between two groups were conducted using independent sample *t*-tests or Mann-Whitney *U*-tests. For multiple group comparisons, we used analysis of variance (ANOVA) or Kruskal-Wallis *H*-tests. Categorical variables were presented as counts (percentages) and analyzed using chi-square tests or Fisher's exact tests. Outliers, defined as observations with absolute *Z*-scores above 3, were treated as missing values. To prevent selective loss of data, missing data were imputed using multiple imputation based on all other available data (mode for categorical variables and mean for continuous variables). *P* values < 0.05 were considered statistically significant.

We identified the confounding factors that needed to be controlled through reviewing previous studies (22–24), conducting relevant analyses (Figure 1B), and performing univariate analysis (Supplementary Table 1). These confounding factors were visualized using a directed acyclic graph (DAG, Figure 1C). Variance inflation factors were utilized to assess multicollinearity among these variables. To generate propensity scores, we used a multinomial logistic regression model, treating mRS scores at different time points or mRS trajectories as the dependent variable and the identified confounding factors as covariates. Using these estimated propensity scores as weights, we constructed an IPTW model. This model allowed us to compare the differences in prevalence between different subgroups in the weighted cohort. Supplementary Figures 1 and 2 illustrated the balance of covariates between different groups before and after IPTW adjustment, evaluating the effect of the adjustment.

GBTM was employed to determine the trajectories of mRS after ICH, assessing the evolution of neurological rehabilitation in survivors. The number (1–4) and shapes (linear, quadratic) of trajectories were selected based on Akaike Information Criterion and Bayesian Information Criterion. The model should satisfy the following criteria: participants in each trajectory group >5%, an average posterior probability >0.7, and the significance of model parameters confirmed by the maximum likelihood estimation method (25). Ultimately, two different rehabilitation trajectories were identified as the best-fit model.

Multivariate logistic regression was performed to explore the association between 3-/6-/12-month mRS scores or rehabilitation trajectories and affective disturbances and cognitive impairment. Model 1 served as the unadjusted model. Adjustments for age, sex, educational level, and marital status were made in Model 2. In the fully adjusted Model 3, additional covariates included alcohol intake, smoking, medical history (diabetes, hypertension, psychiatric history, previous cognitive impairment), hospitalization duration, admission systolic and diastolic blood pressure, GCS, hematoma volume, cerebral hemisphere, ventricular involvement, anatomical distribution, ADL, WML, and encephalatrophy (linear measurements and visual templates).

We conducted sensitivity analyses using E-values to assess the potential impact of unmeasured confounders. Subgroup analyses were stratified by age ( ≤ 57 vs. >57 years), gender, educational level, marital status, hematoma volume ( ≤ 10.3 vs. >10.3 ml), anatomical distribution, presence of WML, frontal ratio ( ≤ 0.32 vs. >0.32), third ventricle Sylvian fissure distance ( ≤ 38.48 vs. >38.48 mm), and the presence of central and cortical brain atrophy.

## Results

### Study participants

Based on the inclusion and exclusion criteria, we obtained a total cohort of 1,692 patients from an initial pool of 4,969 patients. Complete follow-up data were available for 1,563 patients, with a mean follow-up duration of 3.49 ± 0.81 years. Among the 129 patients (7.6%) lost to follow-up, the primary cause was death (75, 58.1%), and 54 (41.9%) were missing at least one item related to cognitive function, depression, or anxiety (Figure 1A).

In our cohort, as detailed in Tables 1 and 2, 915 patients (58.5%) exhibited cognitive impairment, 825 (52.8%) had depression, 616 (39.4%) experienced anxiety, and 658 (42.1%) suffered from both affective disturbances and cognitive impairments. The prevalence were slightly higher than those reported in previous studies. This discrepancy might be attributed to our study population, which exclusively included patients with spontaneous supratentorial ICH and small hematoma volumes (< 30 ml; 10.30, IQR 4.79–17.40 ml).

**Table 1:** Participant characteristics.

Variables	Overall (*n* = 1,563)	Cognitive impairment (*n* = 915)	Depression (*n* = 825)	Anxiety (*n* = 616)	Affective disturbances and cognitive impairment (*n* = 658)
Age, years	57.00 (51.00, 65.00)	59.00 (52.00, 66.00)	58.00 (52.00, 64.00)	59.00 (52.00, 67.00)	59.00 (52.25, 67.00)
Male, *n* (%)	1,022 (65.4)	588 (64.3)	581 (70.4)	373 (60.6)	429 (65.2)
Higher education, *n* (%)	467 (29.9)	255 (27.9)	243 (29.5)	185 (30.0)	185 (28.1)
Marital status, *n* (%)					
Married	1,380 (88.3)	802 (87.7)	737 (89.3)	503 (81.7)	564 (85.7)
Divorced/living alone	98 (6.3)	48 (5.2)	46 (5.6)	55 (8.9)	38 (5.8)
Widowed	85 (5.4)	65 (7.1)	42 (5.1)	58 (9.4)	56 (8.5)
Alcohol intake, *n* (%)	444 (28.4)	240 (26.2)	273 (33.1)	182 (29.5)	192 (29.2)
Smoking, *n* (%)	441 (28.2)	261 (28.5)	273 (33.1)	168 (27.3)	198 (30.1)
Diabetes, *n* (%)	186 (11.9)	98 (10.7)	77 (9.3)	67 (10.9)	65 (9.9)
Hypertension, *n* (%)	1,096 (70.1)	577 (63.1)	578 (70.1)	411 (66.7)	408 (62.0)
Psychiatric history, *n* (%)	532 (34.0)	321 (35.1)	338 (41.0)	242 (39.3)	257 (39.1)
IQCODE (scores 53–63), *n* (%)	322 (20.6)	179 (19.6)	203 (24.6)	98 (15.9)	132 (20.1)
Hospitalization duration, day	9.00 (7.00, 11.00)	8.00 (7.00, 11.00)	9.00 (7.00, 12.00)	9.00 (7.00, 12.00)	9.00 (7.00, 12.00)
Systolic blood pressure, mmHg	165.00 (148.00, 181.00)	159.00 (143.50, 174.00)	163.00 (147.00, 180.00)	162.00 (147.00, 180.00)	158.00 (143.00, 174.00)
Diastolic blood pressure, mmHg	97.00 (86.00, 107.00)	94.00 (85.00, 102.00)	96.00 (86.00, 107.00)	94.00 (84.00, 103.00)	93.00 (84.00, 102.00)
**GCS**, ***n*** **(%)**					
Mild (13–15)	1,181 (75.6)	710 (77.6)	646 (78.3)	480 (77.9)	524 (79.6)
Moderate (9–12)	303 (19.4)	158 (17.3)	148 (17.9)	109 (17.7)	103 (15.7)
Severe (3–8)	79 (5.1)	47 (5.1)	31 (3.8)	27 (4.4)	31 (4.7)
Hematoma volume, ml	10.30 (4.79, 17.40)	8.71 (4.07, 15.93)	12.06 (5.70, 19.50)	9.38 (4.26, 16.57)	9.31 (4.18, 16.79)
Left hematoma, *n* (%)	791 (50.6)	416 (45.5)	397 (48.1)	295 (47.9)	296 (45.0)
Ventricular involvement, *n* (%)	503 (32.2)	270 (29.5)	215 (26.1)	167 (27.1)	167 (25.4)
**Anatomical distribution**, ***n*** **(%)**					
Deep location	1,324 (84.7)	808 (88.3)	694 (84.1)	515 (83.6)	577 (87.7)
Lobar location	239 (15.3)	107 (11.7)	131 (15.9)	101 (16.4)	81 (12.3)
ADL < 95, *n* (%)	700 (44.8)	456 (49.8)	363 (44.0)	278 (45.1)	321 (48.8)

IQCODE, informant questionnaire on cognitive decline in the elderly; GCS, Glasgow Coma scale; ADL, activities of daily living scale.

**Table 2:** Baseline neuroimaging characteristics of the study population.

Variables	Overall (*n* = 1,563)	Cognitive impairment (*n* = 915)	Depression (*n* = 825)	Anxiety (*n* = 616)	Affective disturbances and cognitive impairment (*n* = 658)
White matter lesions, *n* (%)	574 (36.7)	412 (45.0)	306 (37.1)	246 (39.9)	302 (45.9)
Encephalatrophy					
a/b, %	0.32 (0.30, 0.34)	0.32 (0.30, 0.34)	0.32 (0.30, 0.34)	0.32 (0.30, 0.34)	0.32 (0.30, 0.34)
C, mm	38.48 (37.19, 39.80)	38.12 (36.72, 39.30)	38.66 (37.37, 40.11)	38.42 (37.08, 39.74)	38.12 (36.80, 39.49)
Central, *n* (%)	1,047 (67.0)	683 (74.6)	554 (67.2)	429 (69.6)	495 (75.2)
Cortical, *n* (%)	1,042 (66.7)	685 (74.9)	559 (67.8)	460 (74.7)	503 (76.4)
Intracranial volume, ml	1,295.27 (132.20)	1,284.70 (134.31)	1,310.23 (131.55)	1,282.27 (135.28)	1,288.56 (135.04)
Anteroposterior diameter, mm	149.10 (144.70, 153.30)	148.50 (144.20, 152.90)	149.30 (145.40, 153.48)	148.50 (144.30, 152.50)	148.54 (144.40, 152.60)
Transverse diameter, mm	134.46 (130.70, 138.20)	134.00 (130.07, 137.70)	134.58 (131.20, 138.34)	134.19 (130.30, 137.70)	134.10 (130.22, 137.60)

The anteroposterior and transverse intracranial diameters are the maximum distances obtainable from CT scans. Intracranial volume is calculated after 3D Slicer automatically identifies each slice and reconstructs a three-dimensional model to determine the total volume. a/b, frontal ratio; C, third ventricle Sylvian fissure distance.

### Temporal differences in prevalence of specific outcomes based on functional status

**Figure 2:**
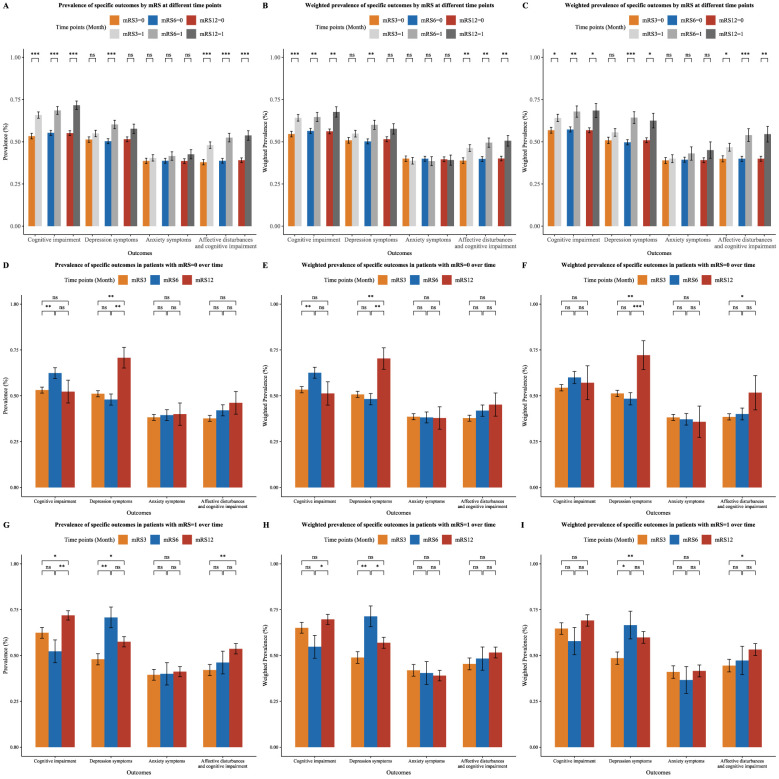
Comparison of outcome prevalence by functional status at various time points. **(A)** Comparison of the unweighted prevalence across different mRS statuses at various time points. **(B)** Comparison of the weighted prevalence across different mRS statuses at various time points based on demographic factors. **(C)** Comparison of the weighted prevalence across different mRS statuses at various time points based on all identified confounding factors. **(D)** Comparison of the unweighted prevalence among those with mRS = 0 at various time points. **(E)** Comparison of the weighted prevalence based on demographic factors among those with mRS = 0 at various time points. **(F)** Comparison of the weighted prevalence based on all identified confounding factors among those with mRS = 0 at various time points. **(G)** Comparison of the unweighted prevalence among those with mRS=1 at various time points. **(H)** Comparison of the weighted prevalence based on demographic factors among those with mRS = 1 at various time points. **(I)** Comparison of the weighted prevalence based on all identified confounding factors among those with mRS = 1 at various time points. mRS, the modified Rankin scale. ns, *P* > 0.05. *P < 0.05. **P < 0.01. ***P < 0.001.

Comparing the prevalence of various symptoms at different time points revealed that patients with favorable neurological recovery (mRS = 0) exhibited significantly lower prevalence (Figures 2A–C). Among these patients, the prevalence of cognitive impairment and anxiety remained relatively stable throughout the rehabilitation period, whereas the prevalence of depression peaked at 12 months of rehabilitation (Figures 2D–F). Patients who didn't achieve favorable rehabilitation outcomes (mRS = 1) showed consistently higher prevalence at all time points, with notable fluctuations over time, particularly in the prevalence of cognitive impairment and depression. However, the overall trend remained unchanged (Figures 2G–I). These findings were further substantiated by weighted analysis.

### Prevalence of specific outcomes by neurological rehabilitation trajectories

**Figure 3:**
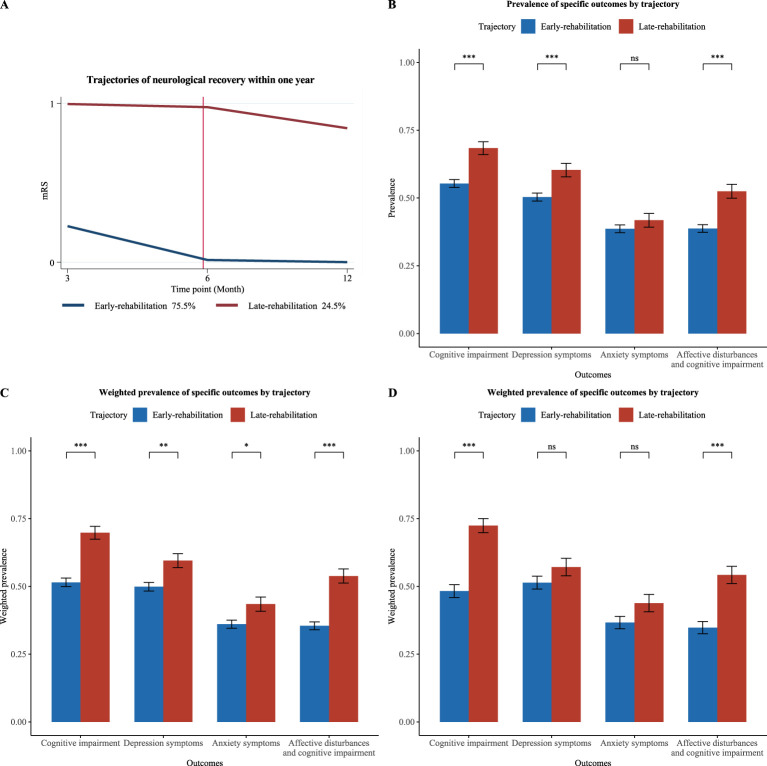
Comparison of outcome prevalence by neurological rehabilitation trajectories. **(A)** Trajectories of neurological rehabilitation within 1 year. **(B)** Comparison of the unweighted prevalence across different trajectories. **(C)** Comparison of the weighted prevalence across different trajectories based on demographic factors. **(D)** Comparison of the weighted prevalence across different trajectories based on all identified confounding factors. mRS, the modified Rankin scale. ns, *P* > 0.05. *P < 0.05. **P < 0.01. ***P < 0.001.

Based on changes in mRS, GBTM identified two trajectory groups of neurological rehabilitation (Figure 3A). The trajectories were labeled as early-rehabilitation (*n* = 1,180, 75.5%) and late-rehabilitation (*n* = 383, 24.5%) based on their rehabilitation duration. The average posterior probabilities for these two trajectories were 0.999 and 0.997, respectively (Supplementary Tables 2–4).

In the IPTW-adjusted analysis, cognitive impairment was significantly more prevalent in the late-rehabilitation group compared to the early-rehabilitation group (Figures 3B–D). Anxiety prevalence showed a statistically significant difference only after adjusting for demographic factors (Model 2, *P* = 0.012, Figure 3C). Conversely, the prevalence of depression didn't exhibit a significant difference after adjusting for all confounding factors (Model 3, *P* = 0.152, Figure 3D).

### Association between specific outcomes and neurological rehabilitation

As shown in Table 3, we conducted both summary analysis and trajectory analysis on the mRS data at three distinct time points. The results are presented in three models (Model 1, Model 2, Model 3), each reflecting different levels of adjustment.

**Table 3:** Association of the modified Rankin scale score (mRS) with affective disturbances and cognitive impairment among intracerebral hemorrhage survivors.

Outcome	Model 1		Model 2		Model 3	
	OR (95%CI)	*P*-value	AOR (95%CI)	*P*-value	AOR (95%CI)	*P*-value
**Cognitive impairment (*****n*** = **915)**						
mRS3	1.42 (1.08, 1.86)	0.012	1.42 (1.08, 1.88)	0.013	1.30 (0.94, 1.81)	0.115
mRS6	0.75 (0.45, 1.26)	0.277	0.68 (0.41, 1.16)	0.154	0.90 (0.49, 1.65)	0.730
mRS12	2.09 (1.25, 3.49)	0.005	1.87 (1.11, 3.15)	0.019	1.44 (0.78, 2.65)	0.243
Late-rehabilitation	1.75 (1.37, 2.24)	< 0.001	1.45 (1.13, 1.87)	0.004	1.40 (1.02, 1.93)	0.036
**Depression symptoms (*****n*** = **825)**						
mRS3	0.87 (0.66, 1.13)	0.302	0.90 (0.69, 1.18)	0.456	0.86 (0.64, 1.16)	0.324
mRS6	2.55 (1.50, 4.45)	< 0.001	2.65 (1.55, 4.64)	< 0.001	2.88 (1.61, 5.27)	< 0.001
mRS12	0.60 (0.35, 1.00)	0.055	0.56 (0.32, 0.95)	0.035	0.71 (0.39, 1.26)	0.242
Late-rehabilitation	1.50 (1.19, 1.90)	< 0.001	1.53 (1.20, 1.95)	< 0.001	1.96 (1.47, 2.62)	< 0.001
**Anxiety symptoms (*****n*** = **616)**						
mRS3	1.01 (0.77, 1.33)	0.938	1.00 (0.75, 1.32)	0.990	1.01 (0.75, 1.36)	0.932
mRS6	0.92 (0.54, 1.54)	0.756	0.82 (0.48, 1.40)	0.475	0.90 (0.51, 1.57)	0.712
mRS12	1.27 (0.76, 2.12)	0.364	1.16 (0.69, 1.97)	0.582	1.17 (0.68, 2.05)	0.575
Late-rehabilitation	1.14 (0.90, 1.44)	0.276	0.94 (0.74, 1.21)	0.652	1.06 (0.80, 1.39)	0.700
**Affective disturbances and cognitive impairment (*****n*** = **658)**						
mRS3	1.17 (0.89, 1.54)	0.247	1.19 (0.90, 1.57)	0.209	1.08 (0.80, 1.47)	0.619
mRS6	1.20 (0.72, 1.99)	0.483	1.11 (0.66, 1.86)	0.692	1.41 (0.80, 2.49)	0.234
mRS12	1.37 (0.83, 2.26)	0.214	1.21 (0.73, 2.00)	0.467	1.10 (0.63, 1.93)	0.734
Late-rehabilitation	1.75 (1.39, 2.21)	< 0.001	1.48 (1.16, 1.88)	0.002	1.62 (1.22, 2.16)	< 0.001

Model 1: unadjusted analysis. Model 2: adjusted for age, sex, educational level and marital status. Model 3: adjusted for age, sex, educational level, marital status, alcohol intake, smoking, medical history (diabetes, hypertension, psychiatric history, dementia), hospitalization duration, admission blood pressure, GCS score, hematoma volume, cerebral hemisphere, ventricular involvement, anatomical distribution, ADL scale score, WML, encephalatrophy (linear measurements and visual templates). mRS, the modified Rankin Scale; OR, odds ratio; CI, confidence interval. P < 0.05 indicates statistical significance.

In the summary analysis, mRS scores at 3 and 12 months were associated with cognitive impairment, but this association disappeared after adjusting for all confounding factors. Across all models, the mRS score at 6 months was significantly associated with depression. However, there was no significant association between mRS scores and anxiety in any of the models. The trajectory analysis, identified using GBTM, revealed that late rehabilitation was significantly associated with both cognitive impairment and depression, but not with anxiety. These associations persisted among patients who experienced concurrent affective and cognitive impairments.

### Impacts of additional factors on specific outcomes across different models

During the summary and trajectory analysis, we identified some notable influencing factors, as presented in Supplementary Tables 5 and 6.

In trajectory analyses, age was associated with cognitive impairment (OR 1.49, 95% CI 1.32–1.67, *P* < 0.001) and anxiety (OR 1.28, 95% CI 1.14–1.44, *P* < 0.001) in Model 2, while it was associated only with depression (OR 1.32, 95% CI 1.12–1.55, *P* < 0.001) in Model 3. In Model 2, males exhibited a heightened risk of depression (OR 1.69, 95% CI 1.36–2.10, *P* < 0.001), whereas females were more predisposed to anxiety (Males: OR 0.75, 95% CI 0.60–0.94, *P* = 0.012). However, after adjusting for all confounding factors (Model 3), sex was solely associated with a risk of anxiety (Males: OR 0.67, 95% CI 0.52–0.87, *P* = 0.003). Regarding marital status, individuals who experienced incomplete marriages exhibited a significantly higher propensity for anxiety symptoms compared to others, with a risk about three times higher. Across all models, we didn't discern any correlation between educational attainment and the specific outcomes.

In the fully adjusted trajectory analysis, we found an intriguing paradox where hematoma volume might protect against cognitive impairment (OR 0.73, 95% CI 0.64–0.83, *P* < 0.001) while increasing the risk of depression (OR 1.70, 95% CI 1.49–1.94, *P* < 0.001). Other protective factors against cognitive impairment included alcohol intake (OR 0.60, 95% CI 0.43–0.83, *P* = 0.002), hypertension (OR 0.39, 95% CI 0.29–0.52, *P* < 0.001), psychiatric history (OR 0.74, 95% CI 0.56–0.98, *P* = 0.035), systolic blood pressure (SBP) at admission (OR 0.44, 95% CI 0.37–0.53, *P* < 0.001), left hematoma (OR 0.69, 95% CI 0.53–0.88, *P* = 0.003), ventricular involvement (OR 0.61, 95% CI 0.47–0.80, *P* < 0.001), and lobar ICH (OR 0.25, 95% CI 0.18–0.36, *P* < 0.001). Consistent with previous research, smoking (OR 1.55, 95% CI 1.11–2.18, *P* = 0.011), ADL (OR 1.71, 95% CI 1.32–2.21, *P* < 0.001), WML (OR 2.17, 95% CI 1.63–2.90, *P* < 0.001), and encephalatrophy, particularly cortical atrophy (OR 1.61, 95% CI 1.16–2.25, *P* = 0.005), were independently associated with an increased risk of cognitive impairment.

Interestingly, previous cognitive impairment appeared to increase the risk of depression (OR 1.74, 95% CI 1.31–2.33, *P* < 0.001), while reducing the risk of anxiety (OR 0.59, 95% CI 0.44–0.79, *P* < 0.001). Additionally, alcohol intake (Depression: OR 1.40, 95% CI 1.03–1.91, *P* = 0.030; Anxiety: OR 1.61, 95% CI 1.19–2.18, *P* = 0.002), psychiatric history (OR 1.67, 95% CI 1.29–2.15, *P* < 0.001; OR 1.45, 95% CI 1.13–1.85, *P* = 0.004), and hospitalization duration (OR 1.19, 95% CI 1.06–1.35, *P* = 0.004; OR 1.16, 95% CI 1.03–1.30, *P* = 0.013) were identified as independent risk factors for both depression and anxiety.

The influence of covariates on related outcomes was also observed in summary analysis.

### Sensitivity and subgroup analysis

In the sensitivity analysis, the E-values for the association between the late-rehabilitation trajectory and cognitive impairment or depression exceeded two. The E-values for the association between mRS at 3 or 12 months and cognitive impairment exceeded two only in Models 1 and 2. For the association between mRS at 6 months and depression, the E-values exceeded two across all models. This suggested that unmeasured confounders would have needed to be strongly associated with both exposure and outcome to fully explain the observed associations. Even after considering the E-values, our results remained robust, indicating that unmeasured confounding factors were unlikely to have substantially altered our main conclusions (Supplementary Figures 3–6).

**Figure 4:**
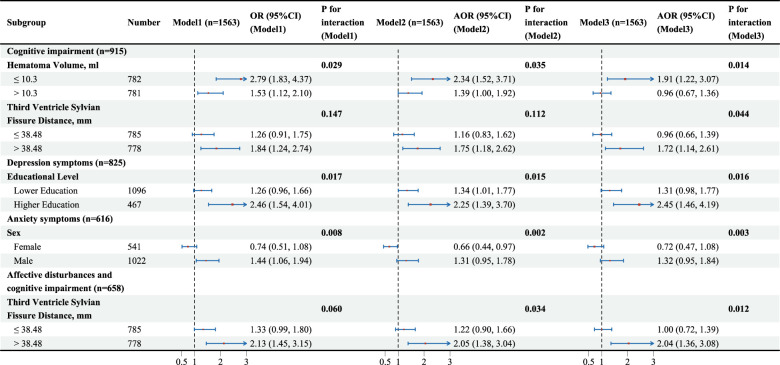
Subgroup analysis of the association between affective and cognitive impairment risk and the late-rehabilitation trajectory. *P* < 0.05 indicates statistical significance.

Figure 4 and Supplementary Tables 7–10 illustrated the risk differences between the late-rehabilitation group and the early-rehabilitation group across different subgroups. In the multivariable-adjusted analysis, the late-rehabilitation group exhibited a higher risk of cognitive impairment in the subgroups with hematoma volume ≤ 10.3 ml and third ventricle Sylvian fissure distance >38.48 mm. Among individuals with a high level of education, those in the late-rehabilitation group had a 1.45-fold increased risk of developing depression. For anxiety, male patients were more likely to have experienced anxiety when undergoing a prolonged rehabilitation period.

## Discussion

This study elucidates the critical impact of rehabilitation trajectories on the prevalence of affective and cognitive impairments in patients following spontaneous supratentorial ICH, particularly in those with small hematomas. Our findings highlight that the trajectory of neurological rehabilitation, as measured by the mRS, significantly influences long-term outcomes, including cognitive function, depression, and anxiety. The results underscore the need for targeted rehabilitation strategies, especially for those on a late-rehabilitation trajectory, to mitigate the risk of persistent neuropsychiatric impairments.

Our analysis identified that patients with favorable early rehabilitation outcomes (mRS ≤ 2 at 3 months) exhibited significantly lower prevalence of cognitive impairment and anxiety throughout the follow-up period. In contrast, those with prolonged or late-rehabilitation trajectories experienced a higher burden of cognitive decline and depressive symptoms. This distinction persisted even after adjusting for confounding variables, suggesting that early and sustained functional rehabilitation is crucial in reducing the long-term neuropsychiatric burden post-ICH.

The association between early rehabilitation and better outcomes aligns with existing literature that emphasizes the importance of early, intensive rehabilitation in stroke recovery (16, 26). However, our study expands on this by using GBTM to provide a more nuanced understanding of how different rehabilitation trajectories affect long-term outcomes. The finding that late rehabilitation is independently associated with increased cognitive impairment and depression, but not anxiety, provides new insights into the temporal dynamics of post-ICH recovery and its neuropsychiatric sequelae.

Age, baseline functional status, and hematoma location emerged as critical predictors of long-term recovery, consistent with previous research (13, 27–30). Older patients and those with baseline disabilities were less likely to experience improvement in functional outcomes, whereas patients with deep ICH had a higher probability of recovery (26). Interestingly, the presence of WML and cortical atrophy, as well as factors such as smoking, were also associated with increased risks of cognitive impairment, suggesting a multifaceted interaction between neurological, lifestyle, and psychological factors.

The paradoxical finding that larger hematoma volumes were protective against cognitive impairment but increased the risk of depression warrants further investigation. This could reflect underlying differences in the mechanisms driving cognitive and affective recovery post-ICH, potentially influenced by the location and extent of brain injury (31, 32). Additionally, the observed associations between alcohol intake, hypertension, and reduced cognitive impairment raise questions about the potential neuroprotective effects of these factors, although the mechanisms remain unclear.

The implications of our study are twofold. Clinically, these results highlight the need for personalized rehabilitation plans that account for the patient's initial rehabilitation trajectory. Such an approach could optimize functional outcomes and reduce the long-term risk of cognitive and affective impairments. Additionally, our findings suggest that future randomized controlled trials should extend follow-up periods beyond the conventional 3-month mark to capture the full spectrum of rehabilitation and its impact on neuropsychiatric health.

Despite its strengths, this study has several limitations. First, the retrospective nature of the analysis may introduce inherent risks of selection bias and information bias. To mitigate these risks, we included all consecutive eligible patients and collected data using standardized protocols; nonetheless, residual confounding cannot be entirely excluded. Second, all patients were recruited from a single hospital, and differences in patient characteristics, clinical management, and follow-up practices across institutions may limit the external validity of our findings. Third, although the modified Rankin Scale (mRS) is widely used and clinically interpretable, it may not fully capture subtle neurological deficits, cognitive changes, mood symptoms, or quality-of-life outcomes. Future prospective studies should incorporate multidimensional assessments, including validated cognitive tests and psychiatric symptom questionnaires. Finally, while we adjusted for a comprehensive range of confounders, residual confounding cannot be entirely ruled out.

## Conclusion

In conclusion, our study underscores the importance of early and sustained neurological rehabilitation in improving long-term cognitive and affective outcomes in ICH survivors. These findings advocate for a more tailored approach to post-ICH care, with an emphasis on early intervention and continuous support, particularly for those at risk of late rehabilitation. Future research should explore the mechanisms underlying these associations and evaluate the efficacy of different rehabilitation strategies in diverse patient populations.

## Data Availability

The original contributions presented in the study are included in the article/Supplementary material, further inquiries can be directed to the corresponding authors.
