# Supplementary material for: Impact of rehabilitation trajectory on affective and cognitive impairment after intracerebral hemorrhage: a cohort study
Source: Front Neurol. 2026 Jun 10;17:1829901. doi: 10.3389/fneur.2026.1829901 (PMC13290636; doi:10.3389/fneur.2026.1829901)
Supplement: Supplementary file 1 [file Table_1.docx]

**SUPPLEMENTARY MATERIALS**

**Impact of rehabilitation trajectory on affective and cognitive impairment after intracerebral hemorrhage**

Qiuyi Jiang, Guangyao Shi, Hongli Zhang, Shouyue Wu, Chunyang Liu, Jian Zhang, Enzhou Lu, Chao Yuan, Yanchao Liang, Lu Wang, Guang Yang

**CONTENTS**

**Supplementary Table 1………………………………………………….……………2**

**Supplementary Table 2…………………………………….…………………………4**

**Supplementary Table 3……………………………………………….………………5**

**Supplementary Table 4………………………………………………….……………6**

**Supplementary Table 5…………………………………………….…………………7**

**Supplementary Table 6…………………………………………………….…………9**

**Supplementary Figure 1…………………………………………….………………11**

**Supplementary Figure 2…………………………………….………………………12**

**Supplementary Figure 3……………………………………………………….……13**

**Supplementary Figure 4………………………………………….…………………14**

**Supplementary Figure 5…………………………………………………….………15**

**Supplementary Figure 6………………………………………………….…………16**

**Supplementary Table 7………………………………………………………...……17**

**Supplementary Table 8…………………………………………………………...…19**

**Supplementary Table 9………………………………………………………...……21**

**Supplementary Table 10…………………………………….…..………………..…23**

**Supplementary Table 1 Univariate logistic regression analysis results of baseline characteristics**

| **Variables** | **Cognitive impairment**  **(n=915)** | | **Depression**  **(n=825)** | | **Anxiety**  **(n=616)** | | **Affective and cognitive**  **impairment (n=658)** | |
| --- | --- | --- | --- | --- | --- | --- | --- | --- |
|  | **OR (95%CI)** | ***P*** | **OR (95%CI)** | ***P*** | **OR (95%CI)** | ***P*** | **OR (95%CI)** | ***P*** |
| Age, years | 1.57 (1.41, 1.75) | <0.001 | 1.09 (0.99, 1.21) | 0.076 | 1.30 (1.17, 1.44) | <0.001 | 1.47 (1.33, 1.64) | <0.001 |
| Male, n (%) | 0.89 (0.72, 1.10) | 0.267 | 1.60 (1.30, 1.98) | <0.001 | 0.70 (0.57, 0.87) | 0.001 | 0.99 (0.80, 1.22) | 0.893 |
| Higher education, n (%) | 0.79 (0.64, 0.99) | 0.039 | 0.96 (0.77, 1.19) | 0.699 | 1.01 (0.81, 1.26) | 0.915 | 0.86 (0.69, 1.08) | 0.194 |
| Marital status, n (%) |  |  |  |  |  |  |  |  |
| Married | 1.00 (reference) | ·· | 1.00 (reference) | ·· | 1.00 (reference) | ·· | 1.00 (reference) | ·· |
| Divorced / living alone | 0.69 (0.46, 1.04) | 0.078 | 0.77 (0.51, 1.16) | 0.216 | 2.23 (1.48, 3.39) | <0.001 | 0.92 (0.60, 1.39) | 0.684 |
| Widowed | 2.34 (1.43, 4.01) | 0.001 | 0.85 (0.55, 1.32) | 0.474 | 3.75 (2.37, 6.07) | <0.001 | 2.79 (1.78, 4.48) | <0.001 |
| Alcohol intake, n (%) | 0.77 (0.62, 0.97) | 0.023 | 1.64 (1.31, 2.05) | <0.001 | 1.10 (0.88, 1.37) | 0.421 | 1.07 (0.85, 1.33) | 0.564 |
| Smoking, n (%) | 1.04 (0.83, 1.30) | 0.747 | 1.68 (1.34, 2.10) | <0.001 | 0.93 (0.74, 1.16) | 0.504 | 1.17 (0.94, 1.46) | 0.160 |
| Diabetes, n (%) | 0.76 (0.56, 1.04) | 0.085 | 0.59 (0.43, 0.81) | 0.001 | 0.85 (0.62, 1.16) | 0.314 | 0.71 (0.51, 0.97) | 0.036 |
| Hypertension, n (%) | 0.42 (0.33, 0.54) | <0.001 | 0.99 (0.80, 1.23) | 0.956 | 0.77 (0.62, 0.96) | 0.018 | 0.51 (0.41, 0.64) | <0.001 |
| Psychiatric history, n (%) | 1.12 (0.90, 1.39) | 0.300 | 1.95 (1.57, 2.41) | <0.001 | 1.47 (1.18, 1.81) | <0.001 | 1.47 (1.19, 1.81) | <0.001 |
| IQCODE (Scores 53-63), n (%) | 0.86 (0.67, 1.10) | 0.228 | 1.70 (1.32, 2.19) | <0.001 | 0.61 (0.47, 0.79) | <0.001 | 0.94 (0.74, 1.21) | 0.652 |
| Hospitalization duration, d | 0.97 (0.88, 1.08) | 0.585 | 1.18 (1.07, 1.31) | 0.001 | 1.05 (0.95, 1.16) | 0.357 | 1.07 (0.97, 1.18) | 0.176 |
| SBP, mmHg | 0.56 (0.50, 0.63) | <0.001 | 0.91 (0.82, 1.00) | 0.050 | 0.89 (0.80, 0.98) | 0.021 | 0.64 (0.57, 0.71) | <0.001 |
| DBP, mmHg | 0.62 (0.55, 0.69) | <0.001 | 0.96 (0.87, 1.06) | 0.423 | 0.74 (0.66, 0.82) | <0.001 | 0.63 (0.56, 0.70) | <0.001 |
| GCS, n (%) |  |  |  |  |  |  |  |  |
| Mild (13-15) | 1.00 (reference) | ·· | 1.00 (reference) | ·· | 1.00 (reference) | ·· | 1.00 (reference) | ·· |
| Moderate (9-12) | 0.98 (0.71, 1.37) | 0.913 | 0.64 (0.46, 0.89) | 0.008 | 0.82 (0.58, 1.15) | 0.258 | 0.86 (0.62, 1.19) | 0.375 |
| Severe (3-8) | 1.29 (0.99, 1.68) | 0.059 | 0.94 (0.72, 1.22) | 0.637 | 1.05 (0.80, 1.38) | 0.728 | 1.31 (1.00, 1.72) | 0.051 |
| Hematoma volume, ml | 0.71 (0.64, 0.79) | <0.001 | 1.45 (1.31, 1.61) | <0.001 | 0.86 (0.78, 0.95) | 0.004 | 0.88 (0.80, 0.98) | 0.015 |
| Left hematoma, n (%) | 0.61 (0.50, 0.74) | <0.001 | 0.81 (0.66, 0.99) | 0.038 | 0.84 (0.68, 1.02) | 0.083 | 0.68 (0.55, 0.83) | <0.001 |
| Ventricular involvement, n (%) | 0.75 (0.60, 0.92) | 0.007 | 0.55 (0.44, 0.68) | <0.001 | 0.68 (0.54, 0.84) | <0.001 | 0.58 (0.46, 0.72) | <0.001 |
| Anatomical location, n (%) |  |  |  |  |  |  |  |  |
| Deep location | 1.00 (reference) | ·· | 1.00 (reference) | ·· | 1.00 (reference) | ·· | 1.00 (reference) | ·· |
| Lobar location | 0.52 (0.39, 0.68) | <0.001 | 1.10 (0.84, 1.45) | 0.495 | 1.15 (0.87, 1.52) | 0.328 | 0.66 (0.50, 0.88) | 0.005 |
| ADL<95, n (%) | 1.64 (1.34, 2.02) | <0.001 | 0.93 (0.77, 1.14) | 0.509 | 1.02 (0.83, 1.25) | 0.825 | 1.32 (1.08, 1.62) | 0.007 |
| White matter lesions, n (%) | 2.46 (1.97, 3.07) | <0.001 | 1.03 (0.84, 1.27) | 0.751 | 1.25 (1.02, 1.55) | 0.034 | 1.97 (1.60, 2.43) | <0.001 |
| Encephalatrophy |  |  |  |  |  |  |  |  |
| a/b, % | 1.36 (1.23, 1.52) | <0.001 | 1.07 (0.97, 1.19) | 0.168 | 1.07 (0.97, 1.18) | 0.193 | 1.24 (1.12, 1.38) | <0.001 |
| C, mm | 0.58 (0.52, 0.65) | <0.001 | 1.29 (1.17, 1.43) | <0.001 | 0.94 (0.85, 1.04) | 0.244 | 0.75 (0.68, 0.83) | <0.001 |
| Central, n (%) | 2.30 (1.85, 2.85) | <0.001 | 1.02 (0.82, 1.25) | 0.883 | 1.22 (0.98, 1.52) | 0.072 | 1.94 (1.56, 2.43) | <0.001 |
| Cortical, n (%) | 2.43 (1.96, 3.01) | <0.001 | 1.11 (0.90, 1.37) | 0.333 | 1.85 (1.48, 2.32) | <0.001 | 2.20 (1.76, 2.76) | <0.001 |
| Intracranial volume, ml | 0.82 (0.74, 0.91) | <0.001 | 1.28 (1.15, 1.41) | <0.001 | 0.85 (0.77, 0.94) | 0.002 | 0.92 (0.83, 1.01) | 0.087 |
| Anteroposterior diameter, mm | 0.84 (0.76, 0.93) | <0.001 | 1.11 (1.00, 1.22) | 0.044 | 0.87 (0.78, 0.96) | 0.005 | 0.86 (0.78, 0.95) | 0.004 |
| Transverse diameter, mm | 0.76 (0.68, 0.84) | <0.001 | 1.14 (1.03, 1.26) | 0.012 | 0.85 (0.77, 0.94) | 0.002 | 0.83 (0.75, 0.92) | <0.001 |

Odds ratios were calculated from univariate logistic regression models. The anteroposterior and transverse intracranial diameters are the maximum distances obtainable from CT scans. Intracranial volume is calculated after 3D Slicer automatically identifies each slice and reconstructs a three-dimensional model to determine the total volume. OR, odds ratio. CI, confidence interval. IQCODE, informant questionnaire on cognitive decline in the elderly. SBP, systolic blood pressure. DBP, diastolic blood pressure. GCS, Glasgow Coma scale. ADL, activities of daily living scale. A/b, frontal ratio. C, third ventricle Sylvian fissure distance. *P<*0.05 indicates statistical significance.

**Supplementary Table 2 The statistics for trajectory models for mRS**

| **Trajectory groups** | **AIC (n=1563)** | **BIC-N-data (n=1563)** | **BIC-n-subjects (n=1563)** |
| --- | --- | --- | --- |
| 1 | -2735.39 | -2748.29 | -2746.10 |
| 2 | -1794.34 | **-1823.38** | -1818.43 |
| 3 | -1779.05 | -1824.22 | **-1816.53** |
| 4 | -1784.05 | -1845.35 | -1834.92 |

AIC, Akaike information criterion. BIC, Bayesian information criterion.

**Supplementary Table 3 The statistics for trajectory models for mRS**

| **Trajectory groups** | **Trajectory shape parameter^1^** | **Group membership (%)** | **Group Avepp** | **Occ** |
| --- | --- | --- | --- | --- |
| 2 | (3 3) | 74.90374 | 0.999 | 265.728 |
|  |  | 25.09626 | 0.997 | 955.924 |
| 2 | (2 2) | 75.55043 | 0.999 | 465.993 |
|  |  | 24.44957 | 0.998 | 1754.594 |
| 2 | (1 1) | 75.55043 | 0.999 | 236.256 |
|  |  | 24.44957 | 0.989 | 281.986 |
| 2 | (3 2) | 74.90375 | 0.999 | 265.728 |
|  |  | 25.09625 | 0.997 | 955.904 |
| 2 | (3 1) | 74.89020 | 0.996 | 94.158 |
|  |  | 25.10980 | 0.998 | 1656.076 |
| 2 | (2 3) | 75.55033 | 0.999 | 465.992 |
|  |  | 24.44967 | 0.998 | 1754.524 |
| 2 | **(2 1)^2^** | 75.47096 | 0.999 | 245.365 |
|  |  | 24.52904 | 0.997 | 1002.846 |
| 2 | (1 3) | 75.73570 | 1.000 | 909.337 |
|  |  | 24.26430 | 0.989 | 283.940 |
| 2 | (1 2) | 75.73569 | 1.000 | 909.345 |
|  |  | 24.26431 | 0.989 | 283.939 |

Avepp, average posterior probability. Occ, odds of correct classification.

^1^Defines the shape parameters of the trajectory groups: 0 = intercept only, 1 = linear, 2 = quadratic.

^2^Represents the best combination of shape parameters based on Bayesian information criterion

**Supplementary Table 4 The Maximum Likelihood Estimates from the Censored Normal Model**

| **Group** | **Parameter** | **Estimate** | **Standard Error** | **T for H0: Parameter=0** | **Prob > \|T\|** |
| --- | --- | --- | --- | --- | --- |
| 1 | Intercept | 8.29211 | 1.87802 | 4.415 | <0.001 |
|  | Linear | -11.9997 | 2.51212 | -4.777 | <0.001 |
|  | Quadratic | 2.49643 | 0.64121 | 3.893 | <0.001 |
|  |  |  |  |  |  |
| 2 | Intercept | 8.10492 | 0.97982 | 8.272 | <0.001 |
|  | Linear | -2.15638 | 0.33722 | -6.395 | <0.001 |
|  |  |  |  |  |  |
| **Group membership** | |  |  |  |  |
|  | Group 1 (%) | 75.47096 | 1.0961 | 68.854 | <0.001 |
|  | Group 2 (%) | 24.52904 | 1.0961 | 22.378 | <0.001 |

**Supplementary Table 5 Multivariate logistic regression analysis results for additional covariates**

| **Variables** | **Cognitive impairment**  **(n=915)** | | **Depression**  **(n=825)** | | **Anxiety**  **(n=616)** | | **Affective and cognitive**  **impairment (n=658)** | |
| --- | --- | --- | --- | --- | --- | --- | --- | --- |
|  | OR (95%CI) | *P* | OR (95%CI) | *P* | OR (95%CI) | *P* | OR (95%CI) | *P* |
| **Model 2** | | | | | | | | |
| Age, years | 1.48 (1.32, 1.66) | <0.001 | 1.09 (0.98, 1.22) | 0.117 | 1.28 (1.14, 1.44) | <0.001 | 1.38 (1.23, 1.55) | <0.001 |
| Male, n (%) | 1.04 (0.83, 1.30) | 0.754 | 1.70 (1.37, 2.11) | <0.001 | 0.75 (0.60, 0.94) | 0.012 | 1.15 (0.92, 1.44) | 0.213 |
| Higher education, n (%) | 0.91 (0.73, 1.15) | 0.431 | 0.94 (0.75, 1.17) | 0.568 | 1.09 (0.86, 1.37) | 0.480 | 0.95 (0.76, 1.19) | 0.660 |
| Marital status, n (%) |  |  |  |  |  |  |  |  |
| Married | 1.00 (reference) | ·· | 1.00 (reference) | ·· | 1.00 (reference) | ·· | 1.00 (reference) | ·· |
| Divorced / living alone | 0.94 (0.61, 1.45) | 0.787 | 0.79 (0.51, 1.20) | 0.270 | 2.83 (1.84, 4.38) | <0.001 | 1.18 (0.76, 1.83) | 0.450 |
| Widowed | 1.33 (0.78, 2.35) | 0.306 | 0.81 (0.51, 1.31) | 0.394 | 2.57 (1.58, 4.27) | <0.001 | 1.79 (1.10, 2.96) | 0.020 |
| **Model 3** | | | | | | | | |
| Age, years | 1.07 (0.90, 1.27) | 0.458 | 1.32 (1.12, 1.55) | <0.001 | 1.10 (0.94, 1.28) | 0.253 | 1.08 (0.92, 1.28) | 0.347 |
| Male, n (%) | 0.94 (0.70, 1.25) | 0.655 | 1.24 (0.95, 1.61) | 0.112 | 0.67 (0.52, 0.87) | 0.003 | 0.91 (0.70, 1.20) | 0.514 |
| Higher education, n (%) | 0.91 (0.69, 1.19) | 0.477 | 0.94 (0.73, 1.20) | 0.601 | 1.00 (0.78, 1.27) | 0.999 | 0.92 (0.71, 1.18) | 0.513 |
| Marital status, n (%) |  |  |  |  |  |  |  |  |
| Married | 1.00 (reference) | ·· | 1.00 (reference) | ·· | 1.00 (reference) | ·· | 1.00 (reference) | ·· |
| Divorced / living alone | 1.02 (0.61, 1.69) | 0.947 | 0.71 (0.44, 1.14) | 0.154 | 3.24 (2.06, 5.15) | <0.001 | 1.26 (0.77, 2.04) | 0.352 |
| Widowed | 1.31 (0.71, 2.50) | 0.391 | 0.68 (0.41, 1.14) | 0.142 | 2.79 (1.67, 4.73) | <0.001 | 1.86 (1.10, 3.19) | 0.023 |
| Alcohol intake, n (%) | 0.60 (0.43, 0.83) | 0.003 | 1.39 (1.02, 1.89) | 0.035 | 1.61 (1.19, 2.18) | 0.002 | 1.08 (0.79, 1.47) | 0.639 |
| Smoking, n (%) | 1.54 (1.10, 2.16) | 0.013 | 1.24 (0.91, 1.69) | 0.168 | 0.71 (0.52, 0.96) | 0.026 | 1.16 (0.85, 1.59) | 0.340 |
| Diabetes, n (%) | 0.76 (0.53, 1.11) | 0.152 | 0.59 (0.42, 0.83) | 0.003 | 0.80 (0.56, 1.12) | 0.196 | 0.73 (0.51, 1.04) | 0.080 |
| Hypertension, n (%) | 0.39 (0.29, 0.52) | <0.001 | 1.16 (0.90, 1.49) | 0.248 | 0.86 (0.67, 1.10) | 0.225 | 0.55 (0.43, 0.71) | <0.001 |
| Psychiatric history, n (%) | 0.75 (0.56, 0.99) | 0.040 | 1.65 (1.28, 2.13) | <0.001 | 1.45 (1.13, 1.86) | 0.003 | 1.13 (0.87, 1.45) | 0.362 |
| IQCODE (scores 53-63) , n (%) | 0.82 (0.61, 1.12) | 0.221 | 1.73 (1.30, 2.31) | <0.001 | 0.59 (0.44, 0.79) | <0.001 | 0.98 (0.73, 1.31) | 0.887 |
| Hospitalization duration, d | 1.09 (0.96, 1.25) | 0.182 | 1.20 (1.06, 1.35) | 0.003 | 1.16 (1.03, 1.31) | 0.013 | 1.20 (1.06, 1.35) | 0.004 |
| SBP, mmHg | 0.44 (0.37, 0.53) | <0.001 | 0.95 (0.81, 1.12) | 0.566 | 1.14 (0.98, 1.34) | 0.097 | 0.69 (0.58, 0.82) | <0.001 |
| DBP, mmHg | 1.15 (0.97, 1.38) | 0.114 | 0.97 (0.83, 1.14) | 0.721 | 0.73 (0.62, 0.85) | <0.001 | 0.88 (0.74, 1.04) | 0.131 |
| GCS, n (%) |  |  |  |  |  |  |  |  |
| Mild (13-15) | 1.00 (reference) | ·· | 1.00 (reference) | ·· | 1.00 (reference) | ·· | 1.00 (reference) | ·· |
| Moderate (9-12) | 1.08 (0.73, 1.61) | 0.714 | 0.50 (0.34, 0.73) | <0.001 | 0.94 (0.64, 1.36) | 0.731 | 0.86 (0.59, 1.25) | 0.443 |
| Severe (3-8) | 1.31 (0.95, 1.80) | 0.097 | 1.03 (0.76, 1.38) | 0.853 | 1.07 (0.79, 1.43) | 0.674 | 1.46 (1.07, 1.98) | 0.016 |
| Hematoma volume, ml | 0.72 (0.63, 0.82) | <0.001 | 1.71 (1.50, 1.96) | <0.001 | 0.91 (0.81, 1.03) | 0.147 | 1.00 (0.88, 1.13) | 0.976 |
| Left hematoma, n (%) | 0.69 (0.54, 0.89) | 0.004 | 0.90 (0.71, 1.13) | 0.351 | 0.90 (0.72, 1.13) | 0.379 | 0.81 (0.64, 1.03) | 0.084 |
| Ventricular involvement, n (%) | 0.61 (0.47, 0.80) | <0.001 | 0.39 (0.30, 0.51) | <0.001 | 0.66 (0.51, 0.84) | <0.001 | 0.45 (0.35, 0.58) | <0.001 |
| Anatomical distribution, n (%) |  |  |  |  |  |  |  |  |
| Deep location | 1.00 (reference) | ·· | 1.00 (reference) | ·· | 1.00 (reference) | ·· | 1.00 (reference) | ·· |
| Lobar location | 0.26 (0.18, 0.37) | <0.001 | 0.78 (0.56, 1.08) | 0.128 | 1.01 (0.74, 1.39) | 0.932 | 0.40 (0.28, 0.56) | <0.001 |
| ADL<95, n (%) | 1.69 (1.31, 2.19) | <0.001 | 0.77 (0.61, 0.97) | 0.030 | 0.79 (0.63, 1.00) | 0.052 | 1.11 (0.87, 1.40) | 0.398 |
| White matter lesions, n (%) | 2.14 (1.61, 2.86) | <0.001 | 1.12 (0.86, 1.46) | 0.387 | 0.99 (0.76, 1.27) | 0.921 | 1.66 (1.28, 2.16) | <0.001 |
| Encephalatrophy |  |  |  |  |  |  |  |  |
| a/b, % | 1.20 (1.04, 1.37) | 0.011 | 1.04 (0.91, 1.18) | 0.586 | 1.07 (0.95, 1.22) | 0.264 | 1.07 (0.94, 1.22) | 0.289 |
| C, mm | 0.67 (0.58, 0.78) | <0.001 | 1.47 (1.29, 1.69) | <0.001 | 1.08 (0.95, 1.23) | 0.237 | 0.91 (0.79, 1.04) | 0.165 |
| Central, n (%) | 1.09 (0.77, 1.55) | 0.607 | 1.02 (0.73, 1.42) | 0.915 | 0.77 (0.55, 1.07) | 0.117 | 1.17 (0.83, 1.64) | 0.367 |
| Cortical, n (%) | 1.59 (1.14, 2.22) | 0.006 | 1.18 (0.85, 1.63) | 0.315 | 2.10 (1.52, 2.91) | <0.001 | 1.60 (1.16, 2.22) | 0.005 |

This table provided the detailed results of the multivariate logistic regression analysis for additional covariates across different outcomes and models. Model2, partially adjusted model. Model3, fully adjusted model. IQCODE, informant questionnaire on cognitive decline in the elderly. SBP, systolic blood pressure. DBP, diastolic blood pressure. GCS, Glasgow Coma scale. ADL, activities of daily living scale. A/b, frontal ratio. C, third ventricle Sylvian fissure distance. OR, odds ratio. CI, confidence interval. *P<*0.05 indicates statistical significance.

**Supplementary Table 6 Logistic regression analysis results based on different trajectories for additional covariates**

| **Variables** | **Cognitive impairment**  **(n=915)** | | **Depression**  **(n=825)** | | **Anxiety**  **(n=616)** | | **Affective and cognitive**  **impairment (n=658)** | |
| --- | --- | --- | --- | --- | --- | --- | --- | --- |
|  | OR (95%CI) | *P* | OR (95%CI) | *P* | OR (95%CI) | *P* | OR (95%CI) | *P* |
| **Model 2** | | | | | | | | |
| Age, years | 1.49 (1.32, 1.67) | <0.001 | 1.09 (0.97, 1.21) | 0.145 | 1.28 (1.14, 1.44) | <0.001 | 1.39 (1.24, 1.56) | <0.001 |
| Male, n (%) | 1.02 (0.82, 1.28) | 0.852 | 1.69 (1.36, 2.10) | <0.001 | 0.75 (0.60, 0.94) | 0.012 | 1.14 (0.92, 1.43) | 0.239 |
| Higher education, n (%) | 0.89 (0.71, 1.12) | 0.320 | 0.95 (0.76, 1.18) | 0.630 | 1.09 (0.86, 1.37) | 0.483 | 0.94 (0.75, 1.18) | 0.593 |
| Marital status, n (%) |  |  |  |  |  |  |  |  |
| Married | 1.00 (reference) | ·· | 1.00 (reference) | ·· | 1.00 (reference) | ·· | 1.00 (reference) | ·· |
| Divorced / living alone | 0.95 (0.62, 1.46) | 0.820 | 0.78 (0.51, 1.20) | 0.263 | 2.83 (1.84, 4.37) | <0.001 | 1.19 (0.76, 1.83) | 0.440 |
| Widowed | 1.33 (0.78, 2.33) | 0.309 | 0.81 (0.50, 1.30) | 0.383 | 2.57 (1.58, 4.27) | <0.001 | 1.79 (1.10, 2.95) | 0.021 |
| **Model 3** | | | | | | | | |
| Age, years | 1.07 (0.90, 1.27) | 0.459 | 1.32 (1.12, 1.55) | <0.001 | 1.09 (0.93, 1.28) | 0.262 | 1.08 (0.92, 1.28) | 0.343 |
| Male, n (%) | 0.92 (0.69, 1.23) | 0.581 | 1.24 (0.96, 1.61) | 0.105 | 0.67 (0.52, 0.87) | 0.003 | 0.91 (0.69, 1.19) | 0.489 |
| Higher education, n (%) | 0.90 (0.69, 1.18) | 0.433 | 0.94 (0.74, 1.20) | 0.630 | 1.00 (0.78, 1.27) | 0.994 | 0.92 (0.71, 1.18) | 0.494 |
| Marital status, n (%) |  |  |  |  |  |  |  |  |
| Married | 1.00 (reference) | ·· | 1.00 (reference) | ·· | 1.00 (reference) | ·· | 1.00 (reference) | ·· |
| Divorced / living alone | 1.01 (0.61, 1.68) | 0.964 | 0.71 (0.44, 1.13) | 0.145 | 3.24 (2.06, 5.15) | <0.001 | 1.26 (0.77, 2.04) | 0.349 |
| Widowed | 1.31 (0.72, 2.48) | 0.392 | 0.69 (0.41, 1.14) | 0.146 | 2.79 (1.68, 4.73) | <0.001 | 1.86 (1.10, 3.20) | 0.022 |
| Alcohol intake, n (%) | 0.60 (0.43, 0.83) | 0.002 | 1.40 (1.03, 1.91) | 0.030 | 1.61 (1.19, 2.18) | 0.002 | 1.08 (0.79, 1.47) | 0.642 |
| Smoking, n (%) | 1.55 (1.11, 2.18) | 0.011 | 1.23 (0.90, 1.67) | 0.187 | 0.71 (0.52, 0.96) | 0.026 | 1.17 (0.85, 1.59) | 0.332 |
| Diabetes, n (%) | 0.77 (0.53, 1.11) | 0.160 | 0.60 (0.42, 0.84) | 0.003 | 0.80 (0.56, 1.12) | 0.194 | 0.73 (0.51, 1.04) | 0.084 |
| Hypertension, n (%) | 0.39 (0.29, 0.52) | <0.001 | 1.16 (0.90, 1.49) | 0.263 | 0.86 (0.67, 1.10) | 0.231 | 0.55 (0.43, 0.71) | <0.001 |
| Psychiatric history, n (%) | 0.74 (0.56, 0.98) | 0.035 | 1.67 (1.29, 2.15) | <0.001 | 1.45 (1.13, 1.85) | 0.004 | 1.12 (0.87, 1.45) | 0.372 |
| IQCODE (Scores 53-63), n (%) | 0.82 (0.60, 1.11) | 0.195 | 1.74 (1.31, 2.33) | <0.001 | 0.59 (0.44, 0.79) | <0.001 | 0.98 (0.73, 1.30) | 0.879 |
| Hospitalization duration, d | 1.10 (0.97, 1.26) | 0.146 | 1.19 (1.06, 1.35) | 0.004 | 1.16 (1.03, 1.30) | 0.013 | 1.20 (1.06, 1.35) | 0.004 |
| SBP, mmHg | 0.44 (0.37, 0.53) | <0.001 | 0.96 (0.82, 1.13) | 0.607 | 1.14 (0.97, 1.33) | 0.105 | 0.69 (0.58, 0.81) | <0.001 |
| DBP, mmHg | 1.16 (0.97, 1.39) | 0.104 | 0.97 (0.82, 1.14) | 0.692 | 0.73 (0.62, 0.86) | <0.001 | 0.88 (0.74, 1.04) | 0.133 |
| GCS, n (%) |  |  |  |  |  |  |  |  |
| Mild (13-15) | 1.00 (reference) | ·· | 1.00 (reference) | ·· | 1.00 (reference) | ·· | 1.00 (reference) | ·· |
| Moderate (9-12) | 1.10 (0.74, 1.63) | 0.651 | 0.49 (0.33, 0.72) | <0.001 | 0.94 (0.64, 1.36) | 0.738 | 0.86 (0.59, 1.26) | 0.449 |
| Severe (3-8) | 1.28 (0.93, 1.76) | 0.130 | 1.05 (0.78, 1.41) | 0.751 | 1.06 (0.79, 1.43) | 0.684 | 1.45 (1.07, 1.97) | 0.017 |
| Hematoma volume, ml | 0.73 (0.64, 0.83) | <0.001 | 1.70 (1.49, 1.94) | <0.001 | 0.91 (0.81, 1.03) | 0.143 | 1.00 (0.88, 1.14) | 0.996 |
| Left hematoma, n (%) | 0.69 (0.53, 0.88) | 0.003 | 0.90 (0.71, 1.13) | 0.354 | 0.90 (0.72, 1.13) | 0.384 | 0.81 (0.64, 1.03) | 0.081 |
| Ventricular involvement, n (%) | 0.61 (0.47, 0.80) | <0.001 | 0.40 (0.31, 0.51) | <0.001 | 0.66 (0.51, 0.84) | <0.001 | 0.45 (0.35, 0.58) | <0.001 |
| Anatomical distribution, n (%) |  |  |  |  |  |  |  |  |
| Deep location | 1.00 (reference) | ·· | 1.00 (reference) | ·· | 1.00 (reference) | ·· | 1.00 (reference) | ·· |
| Lobar location | 0.25 (0.18, 0.36) | <0.001 | 0.79 (0.57, 1.10) | 0.164 | 1.01 (0.74, 1.39) | 0.933 | 0.39 (0.28, 0.55) | <0.001 |
| ADL<95, n (%) | 1.71 (1.32, 2.21) | <0.001 | 0.76 (0.60, 0.97) | 0.024 | 0.80 (0.63, 1.00) | 0.053 | 1.11 (0.87, 1.41) | 0.393 |
| White matter lesions, n (%) | 2.17 (1.63, 2.90) | <0.001 | 1.11 (0.85, 1.44) | 0.443 | 0.99 (0.77, 1.28) | 0.936 | 1.66 (1.28, 2.16) | <0.001 |
| Encephalatrophy |  |  |  |  |  |  |  |  |
| a/b, % | 1.20 (1.05, 1.38) | 0.009 | 1.03 (0.91, 1.17) | 0.606 | 1.07 (0.95, 1.21) | 0.268 | 1.07 (0.94, 1.22) | 0.291 |
| C, mm | 0.66 (0.57, 0.77) | <0.001 | 1.48 (1.30, 1.70) | <0.001 | 1.08 (0.95, 1.23) | 0.240 | 0.91 (0.79, 1.04) | 0.151 |
| Central, n (%) | 1.09 (0.77, 1.54) | 0.620 | 1.01 (0.73, 1.42) | 0.935 | 0.77 (0.55, 1.07) | 0.118 | 1.17 (0.83, 1.64) | 0.363 |
| Cortical, n (%) | 1.61 (1.16, 2.25) | 0.005 | 1.17 (0.85, 1.61) | 0.342 | 2.11 (1.53, 2.93) | <0.001 | 1.61 (1.16, 2.23) | 0.004 |

This table provided the detailed results of the logistic regression analysis based on different trajectories for additional covariates across different outcomes and models. Model2, partially adjusted model. Model3, fully adjusted model. IQCODE, informant questionnaire on cognitive decline in the elderly. SBP, systolic blood pressure. DBP, diastolic blood pressure. GCS, Glasgow Coma scale. ADL, activities of daily living scale. A/b, frontal ratio. C, third ventricle Sylvian fissure distance. OR, odds ratio. CI, confidence interval. *P<*0.05 indicates statistical significance.

**Supplementary Figure 1 The results of covariates balance after propensity score-based inverse probability of treatment weighting in model2.** The balance results between subgroups achieving mRS=0 at different time points (A). The balance results between subgroups achieving mRS=1 at different time points (B). The balance results between subgroups with mRS=0 and mRS=1 at different time points (C). The balance results of different trajectories (D). Model2, partially adjusted model. mRS, the modified Rankin Scale. IQCODE, informant questionnaire on cognitive decline in the elderly. GCS, Glasgow Coma scale. ADL, activities of daily living scale. Absolute standardized mean differences <0.1 indicates good covariate balance.

**Supplementary Figure 2 The results of covariates balance after propensity score-based inverse probability of treatment weighting in model3.** The balance results between subgroups achieving mRS=0 at different time points (A). The balance results between subgroups achieving mRS=1 at different time points (B). The balance results between subgroups with mRS=0 and mRS=1 at different time points (C). The balance results of different trajectories (D). Model3, fully adjusted model. mRS, the modified Rankin Scale. IQCODE, informant questionnaire on cognitive decline in the elderly. GCS, Glasgow Coma scale. ADL, activities of daily living scale. Absolute standardized mean differences <0.1 indicates good covariate balance.

**Supplementary Figure 3 Curves of the sensitivity analysis for unobserved confounders with cognitive impairment.** RR_UD_, risk ratio for confounder-outcome relationship. RR_EU_, risk ratio for exposure-confounder relationship. mRS, the modified Rankin Scale. Model1, unadjusted model. Model2, partially adjusted model. Model3, fully adjusted model.

**Supplementary Figure 4 Curves of the sensitivity analysis for unobserved confounders with depression symptoms.** RR_UD_, risk ratio for confounder-outcome relationship. RR_EU_, risk ratio for exposure-confounder relationship. mRS, the modified Rankin Scale. Model1, unadjusted model. Model2, partially adjusted model. Model3, fully adjusted model.

**Supplementary Figure 5 Curves of the sensitivity analysis for unobserved confounders with anxiety symptoms.** RR_UD_, risk ratio for confounder-outcome relationship. RR_EU_, risk ratio for exposure-confounder relationship. mRS, the modified Rankin Scale. Model1, unadjusted model. Model2, partially adjusted model. Model3, fully adjusted model.

**Supplementary Figure 6 Curves of the sensitivity analysis for unobserved confounders with affective disturbances and cognitive impairment.** RR_UD_, risk ratio for confounder-outcome relationship. RR_EU_, risk ratio for exposure-confounder relationship. mRS, the modified Rankin Scale. Model1, unadjusted model. Model2, partially adjusted model. Model3, fully adjusted model.

**Supplementary Table 7 Subgroup analysis for the cognitive impairment risk according to trajectories of mRS**

| **Subgroup** | **Model 1** | | **Model 2** | | **Model 3** | |
| --- | --- | --- | --- | --- | --- | --- |
|  | OR (95%CI) | *P* for interaction | OR (95%CI) | *P* for interaction | OR (95%CI) | *P* for interaction |
| Age, years |  | 0.516 |  | 0.438 |  | 0.544 |
| ≤ 57 | 1.71 (1.19, 2.47) |  | 1.74 (1.21, 2.52) |  | 1.51 (1.02, 2.25) |  |
| > 57 | 1.45 (1.04, 2.05) |  | 1.38 (0.98, 1.96) |  | 1.08 (0.74, 1.59) |  |
| Sex |  | 0.239 |  | 0.233 |  | 0.274 |
| Female | 2.10 (1.41, 3.17) |  | 1.81 (1.19, 2.79) |  | 1.44 (0.90, 2.32) |  |
| Male | 1.55 (1.14, 2.11) |  | 1.41 (1.03, 1.93) |  | 1.16 (0.83, 1.63) |  |
| Educational level |  | 0.977 |  | 0.851 |  | 0.737 |
| Lower education | 1.73 (1.30, 2.32) |  | 1.55 (1.16, 2.09) |  | 1.33 (0.97, 1.84) |  |
| Higher education | 1.72 (1.09, 2.76) |  | 1.46 (0.91, 2.38) |  | 1.10 (0.65, 1.87) |  |
| Marital status |  | 0.757 |  | 0.842 |  | 0.785 |
| Married | 1.70 (1.31, 2.22) |  | 1.53 (1.18, 2.01) |  | 1.27 (0.95, 1.70) |  |
| Divorced / living alone | 1.19 (0.45, 3.17) |  | 1.39 (0.49, 4.03) |  | 1.68 (0.52, 5.71) |  |
| Widowed | 1.92 (0.69, 5.68) |  | 1.88 (0.67, 5.62) |  | 1.27 (0.33, 4.91) |  |
| Hematoma volume, ml |  | 0.026 |  | 0.035 |  | 0.014 |
| ≤ 10.3 | 2.79 (1.83, 4.37) |  | 2.34 (1.52, 3.71) |  | 1.91 (1.22, 3.07) |  |
| > 10.3 | 1.53 (1.12, 2.10) |  | 1.39 (1.00, 1.92) |  | 0.96 (0.67, 1.36) |  |
| Anatomical distribution |  | 0.975 |  | 0.625 |  | 0.549 |
| Deep location | 1.66 (1.28, 2.17) |  | 1.41 (1.08, 1.85) |  | 1.23 (0.92, 1.65) |  |
| Lobar location | 1.68 (0.81, 3.55) |  | 1.34 (0.61, 2.95) |  | 1.23 (0.54, 2.82) |  |
| White matter lesions |  | 0.720 |  | 0.741 |  | 0.954 |
| No | 1.43 (1.03, 1.99) |  | 1.36 (0.97, 1.90) |  | 1.35 (0.95, 1.92) |  |
| Yes | 1.57 (1.06, 2.34) |  | 1.45 (0.97, 2.19) |  | 1.13 (0.73, 1.76) |  |
| Frontal Ratio, % |  | 0.973 |  | 0.993 |  | 0.677 |
| ≤ 0.32 | 1.62 (1.14, 2.32) |  | 1.44 (1.00, 2.07) |  | 1.35 (0.92, 1.99) |  |
| > 0.32 | 1.61 (1.14, 2.29) |  | 1.44 (1.01, 2.07) |  | 1.12 (0.76, 1.66) |  |
| Third Ventricle Sylvian Fissure Distance, mm | | 0.147 |  | 0.112 |  | 0.044 |
| ≤ 38.48 | 1.26 (0.91, 1.75) |  | 1.16 (0.83, 1.62) |  | 0.96 (0.66, 1.39) |  |
| > 38.48 | 1.84 (1.24, 2.74) |  | 1.75 (1.18, 2.62) |  | 1.72 (1.14, 2.61) |  |
| Encephalatrophy (Central) |  | 0.150 |  | 0.187 |  | 0.533 |
| No | 1.21 (0.77, 1.91) |  | 1.13 (0.71, 1.80) |  | 1.06 (0.65, 1.73) |  |
| Yes | 1.81 (1.34, 2.45) |  | 1.65 (1.22, 2.25) |  | 1.30 (0.94, 1.82) |  |
| Encephalatrophy (Cortical) |  | 0.223 |  | 0.226 |  | 0.373 |
| No | 1.34 (0.88, 2.06) |  | 1.17 (0.75, 1.81) |  | 1.00 (0.63, 1.59) |  |
| Yes | 1.87 (1.37, 2.56) |  | 1.73 (1.27, 2.39) |  | 1.35 (0.96, 1.91) |  |

Model 1: unadjusted analysis. Model 2: adjusted for age, sex, educational level and marital status. Model 3: adjusted for age, sex, educational level, marital status, hematoma volume, anatomical distribution, white matter lesions, encephalatrophy (linear measurements and visual templates). mRS, the modified Rankin Scale. OR, odds ratio. CI, confidence interval. *P*<0.05 indicates statistical significance.

**Supplementary Table 8 Subgroup analysis for the depression risk according to trajectories of mRS**

| **Subgroup** | **Model 1** | | **Model 2** | | **Model 3** | |
| --- | --- | --- | --- | --- | --- | --- |
|  | OR (95%CI) | *P* for interaction | OR (95%CI) | *P* for interaction | OR (95%CI) | *P* for interaction |
| Age, years |  | 0.898 |  | 0.895 |  | 0.804 |
| ≤ 57 | 1.49 (1.04, 2.13) |  | 1.57 (1.09, 2.28) |  | 1.60 (1.09, 2.38) |  |
| > 57 | 1.44 (1.05, 1.98) |  | 1.50 (1.09, 2.08) |  | 1.52 (1.08, 2.15) |  |
| Sex |  | 0.435 |  | 0.388 |  | 0.327 |
| Female | 1.77 (1.21, 2.57) |  | 1.68 (1.15, 2.47) |  | 1.60 (1.06, 2.43) |  |
| Male | 1.46 (1.08, 1.98) |  | 1.44 (1.06, 1.97) |  | 1.49 (1.08, 2.08) |  |
| Educational level |  | 0.015 |  | 0.015 |  | 0.016 |
| Lower education | 1.26 (0.96, 1.66) |  | 1.34 (1.01, 1.77) |  | 1.31 (0.98, 1.77) |  |
| Higher education | 2.46 (1.54, 4.01) |  | 2.25 (1.39, 3.70) |  | 2.45 (1.46, 4.19) |  |
| Marital status |  | 0.637 |  | 0.667 |  | 0.476 |
| Married | 1.49 (1.15, 1.92) |  | 1.50 (1.16, 1.95) |  | 1.53 (1.17, 2.02) |  |
| Divorced / living alone | 1.32 (0.50, 3.52) |  | 1.18 (0.43, 3.25) |  | 1.00 (0.32, 3.12) |  |
| Widowed | 2.25 (0.95, 5.46) |  | 2.42 (1.00, 6.08) |  | 2.57 (0.92, 7.61) |  |
| Hematoma volume, ml |  | 0.319 |  | 0.361 |  | 0.293 |
| ≤ 10.3 | 1.24 (0.88, 1.76) |  | 1.22 (0.85, 1.75) |  | 1.31 (0.90, 1.92) |  |
| > 10.3 | 1.59 (1.15, 2.21) |  | 1.64 (1.17, 2.31) |  | 1.81 (1.27, 2.59) |  |
| Anatomical distribution |  | 0.866 |  | 0.939 |  | 0.859 |
| Deep location | 1.51 (1.18, 1.94) |  | 1.56 (1.21, 2.02) |  | 1.55 (1.18, 2.04) |  |
| Lobar location | 1.61 (0.77, 3.53) |  | 1.56 (0.70, 3.63) |  | 1.71 (0.73, 4.23) |  |
| White matter lesions |  | 0.439 |  | 0.389 |  | 0.471 |
| No | 1.66 (1.19, 2.32) |  | 1.81 (1.29, 2.56) |  | 1.82 (1.27, 2.61) |  |
| Yes | 1.37 (0.97, 1.94) |  | 1.35 (0.95, 1.92) |  | 1.30 (0.90, 1.89) |  |
| Frontal Ratio, % |  | 0.748 |  | 0.643 |  | 0.486 |
| ≤ 0.32 | 1.41 (1.00, 2.00) |  | 1.46 (1.03, 2.10) |  | 1.42 (0.98, 2.06) |  |
| > 0.32 | 1.53 (1.10, 2.12) |  | 1.55 (1.11, 2.18) |  | 1.73 (1.21, 2.50) |  |
| Third Ventricle Sylvian Fissure Distance, mm | | 0.153 |  | 0.162 |  | 0.124 |
| ≤ 38.48 | 1.46 (1.09, 1.97) |  | 1.49 (1.10, 2.02) |  | 1.29 (0.93, 1.79) |  |
| > 38.48 | 2.12 (1.41, 3.27) |  | 2.13 (1.40, 3.31) |  | 2.11 (1.37, 3.31) |  |
| Encephalatrophy (Central) |  | 0.238 |  | 0.224 |  | 0.319 |
| No | 1.92 (1.20, 3.12) |  | 1.99 (1.23, 3.28) |  | 1.90 (1.15, 3.18) |  |
| Yes | 1.38 (1.05, 1.82) |  | 1.44 (1.09, 1.91) |  | 1.46 (1.08, 1.98) |  |
| Encephalatrophy (Cortical) |  | 0.903 |  | 0.957 |  | 0.870 |
| No | 1.46 (0.95, 2.25) |  | 1.55 (0.99, 2.45) |  | 1.54 (0.96, 2.49) |  |
| Yes | 1.50 (1.14, 1.99) |  | 1.54 (1.16, 2.06) |  | 1.58 (1.17, 2.15) |  |

Model 1: unadjusted analysis. Model 2: adjusted for age, sex, educational level and marital status. Model 3: adjusted for age, sex, educational level, marital status, hematoma volume, anatomical distribution, white matter lesions, encephalatrophy (linear measurements and visual templates). mRS, the modified Rankin Scale. OR, odds ratio. CI, confidence interval. *P*<0.05 indicates statistical significance.

**Supplementary Table 9 Subgroup analysis for the anxiety risk according to trajectories of mRS**

| **Subgroup** | **Model 1** | | **Model 2** | | **Model 3** | |
| --- | --- | --- | --- | --- | --- | --- |
|  | OR (95%CI) | *P* for interaction | OR (95%CI) | *P* for interaction | OR (95%CI) | *P* for interaction |
| Age, years |  | 0.347 |  | 0.395 |  | 0.361 |
| ≤ 57 | 0.94 (0.64, 1.35) |  | 0.87 (0.59, 1.26) |  | 0.96 (0.64, 1.44) |  |
| > 57 | 1.18 (0.87, 1.61) |  | 1.11 (0.80, 1.53) |  | 1.11 (0.79, 1.56) |  |
| Sex |  | 0.007 |  | 0.002 |  | 0.003 |
| Female | 0.74 (0.51, 1.08) |  | 0.66 (0.44, 0.97) |  | 0.72 (0.47, 1.08) |  |
| Male | 1.44 (1.06, 1.94) |  | 1.31 (0.95, 1.78) |  | 1.32 (0.95, 1.84) |  |
| Educational level |  | 0.758 |  | 0.682 |  | 0.752 |
| Lower education | 1.12 (0.85, 1.47) |  | 0.96 (0.72, 1.28) |  | 1.04 (0.77, 1.41) |  |
| Higher education | 1.21 (0.77, 1.90) |  | 1.09 (0.67, 1.74) |  | 1.01 (0.61, 1.66) |  |
| Marital status |  | 0.645 |  | 0.732 |  | 0.697 |
| Married | 1.10 (0.85, 1.43) |  | 1.02 (0.79, 1.33) |  | 1.06 (0.80, 1.40) |  |
| Divorced / living alone | 0.83 (0.31, 2.20) |  | 1.06 (0.38, 3.02) |  | 1.07 (0.35, 3.30) |  |
| Widowed | 0.75 (0.30, 1.89) |  | 0.76 (0.29, 1.94) |  | 1.15 (0.36, 3.78) |  |
| Hematoma volume, ml |  | 0.927 |  | 0.859 |  | 0.918 |
| ≤ 10.3 | 1.18 (0.83, 1.68) |  | 0.95 (0.65, 1.37) |  | 1.00 (0.68, 1.46) |  |
| > 10.3 | 1.16 (0.84, 1.59) |  | 1.06 (0.76, 1.47) |  | 1.06 (0.74, 1.50) |  |
| Anatomical distribution |  | 0.875 |  | 0.694 |  | 0.642 |
| Deep location | 1.16 (0.91, 1.49) |  | 1.00 (0.77, 1.30) |  | 1.08 (0.82, 1.41) |  |
| Lobar location | 1.09 (0.52, 2.27) |  | 0.91 (0.39, 2.03) |  | 0.83 (0.35, 1.91) |  |
| White matter lesions |  | 0.483 |  | 0.512 |  | 0.604 |
| No | 1.00 (0.71, 1.40) |  | 0.90 (0.64, 1.27) |  | 1.00 (0.70, 1.44) |  |
| Yes | 1.19 (0.84, 1.68) |  | 1.07 (0.74, 1.53) |  | 1.04 (0.71, 1.51) |  |
| Frontal Ratio, % |  | 0.237 |  | 0.310 |  | 0.295 |
| ≤ 0.32 | 0.98 (0.69, 1.38) |  | 0.89 (0.61, 1.27) |  | 0.93 (0.64, 1.35) |  |
| > 0.32 | 1.30 (0.94, 1.81) |  | 1.12 (0.79, 1.57) |  | 1.13 (0.79, 1.61) |  |
| Third Ventricle Sylvian Fissure Distance, mm | | 0.402 |  | 0.253 |  | 0.206 |
| ≤ 38.48 | 1.03 (0.76, 1.40) |  | 0.90 (0.66, 1.23) |  | 0.87 (0.62, 1.22) |  |
| > 38.48 | 1.28 (0.86, 1.88) |  | 1.20 (0.80, 1.80) |  | 1.32 (0.86, 1.99) |  |
| Encephalatrophy (Central) |  | 0.214 |  | 0.347 |  | 0.406 |
| No | 0.86 (0.52, 1.38) |  | 0.80 (0.49, 1.31) |  | 0.86 (0.51, 1.43) |  |
| Yes | 1.21 (0.92, 1.59) |  | 1.06 (0.80, 1.40) |  | 1.09 (0.81, 1.48) |  |
| Encephalatrophy (Cortical) |  | 0.933 |  | 0.951 |  | 0.945 |
| No | 1.07 (0.67, 1.69) |  | 1.05 (0.64, 1.69) |  | 1.12 (0.67, 1.83) |  |
| Yes | 1.10 (0.83, 1.45) |  | 0.97 (0.73, 1.30) |  | 1.00 (0.74, 1.36) |  |

Model 1: unadjusted analysis. Model 2: adjusted for age, sex, educational level and marital status. Model 3: adjusted for age, sex, educational level, marital status, hematoma volume, anatomical distribution, white matter lesions, encephalatrophy (linear measurements and visual templates). mRS, the modified Rankin Scale. OR, odds ratio. CI, confidence interval. *P*<0.05 indicates statistical significance.

**Supplementary Table 10 Subgroup analysis for affective disturbances and cognitive impairment risk according to trajectories of mRS**

| **Subgroup** | **Model 1** | | **Model 2** | | **Model 3** | |
| --- | --- | --- | --- | --- | --- | --- |
|  | OR (95%CI) | *P* for interaction | OR (95%CI) | *P* for interaction | OR (95%CI) | *P* for interaction |
| Age, years |  | 0.583 |  | 0.472 |  | 0.558 |
| ≤ 57 | 1.74 (1.21, 2.49) |  | 1.74 (1.21, 2.51) |  | 1.63 (1.11, 2.40) |  |
| > 57 | 1.52 (1.11, 2.08) |  | 1.45 (1.06, 2.00) |  | 1.21 (0.87, 1.70) |  |
| Sex |  | 0.936 |  | 0.858 |  | 0.813 |
| Female | 1.73 (1.19, 2.52) |  | 1.50 (1.02, 2.22) |  | 1.31 (0.86, 2.00) |  |
| Male | 1.76 (1.31, 2.38) |  | 1.60 (1.18, 2.17) |  | 1.38 (1.00, 1.90) |  |
| Educational level |  | 0.386 |  | 0.410 |  | 0.492 |
| Lower education | 1.63 (1.24, 2.14) |  | 1.49 (1.13, 1.96) |  | 1.34 (1.00, 1.80) |  |
| Higher education | 2.06 (1.31, 3.25) |  | 1.79 (1.12, 2.87) |  | 1.47 (0.89, 2.42) |  |
| Marital status |  | 0.265 |  | 0.348 |  | 0.264 |
| Married | 1.74 (1.35, 2.23) |  | 1.62 (1.25, 2.09) |  | 1.40 (1.07, 1.84) |  |
| Divorced / living alone | 0.74 (0.26, 2.00) |  | 0.85 (0.28, 2.44) |  | 0.98 (0.30, 3.12) |  |
| Widowed | 1.76 (0.71, 4.49) |  | 1.84 (0.73, 4.78) |  | 1.69 (0.56, 5.24) |  |
| Hematoma volume, ml |  | 0.434 |  | 0.536 |  | 0.425 |
| ≤ 10.3 | 2.00 (1.41, 2.85) |  | 1.71 (1.19, 2.46) |  | 1.52 (1.04, 2.22) |  |
| > 10.3 | 1.66 (1.21, 2.27) |  | 1.52 (1.10, 2.10) |  | 1.20 (0.85, 1.69) |  |
| Anatomical distribution |  | 0.739 |  | 0.848 |  | 0.882 |
| Deep location | 1.68 (1.31, 2.15) |  | 1.48 (1.15, 1.91) |  | 1.35 (1.03, 1.77) |  |
| Lobar location | 1.91 (0.91, 4.00) |  | 1.52 (0.67, 3.35) |  | 1.46 (0.62, 3.33) |  |
| White matter lesions |  | 0.724 |  | 0.643 |  | 0.509 |
| No | 1.61 (1.16, 2.23) |  | 1.56 (1.11, 2.17) |  | 1.60 (1.13, 2.27) |  |
| Yes | 1.48 (1.05, 2.09) |  | 1.36 (0.96, 1.94) |  | 1.14 (0.78, 1.65) |  |
| Frontal Ratio, % |  | 0.159 |  | 0.161 |  | 0.190 |
| ≤ 0.32 | 1.39 (0.98, 1.96) |  | 1.27 (0.89, 1.81) |  | 1.16 (0.80, 1.68) |  |
| > 0.32 | 1.95 (1.41, 2.70) |  | 1.75 (1.26, 2.45) |  | 1.55 (1.09, 2.21) |  |
| Third Ventricle Sylvian Fissure Distance, mm | | 0.060 |  | 0.034 |  | 0.012 |
| ≤ 38.48 | 1.33 (0.99, 1.80) |  | 1.22 (0.90, 1.66) |  | 1.00 (0.72, 1.39) |  |
| > 38.48 | 2.13 (1.45, 3.15) |  | 2.05 (1.38, 3.04) |  | 2.04 (1.36, 3.08) |  |
| Encephalatrophy (Central) |  | 0.454 |  | 0.600 |  | 0.951 |
| No | 1.40 (0.86, 2.24) |  | 1.37 (0.84, 2.20) |  | 1.32 (0.79, 2.17) |  |
| Yes | 1.72 (1.31, 2.26) |  | 1.58 (1.20, 2.09) |  | 1.34 (1.00, 1.81) |  |
| Encephalatrophy (Cortical) |  | 0.565 |  | 0.645 |  | 0.813 |
| No | 1.50 (0.95, 2.34) |  | 1.41 (0.89, 2.23) |  | 1.28 (0.79, 2.06) |  |
| Yes | 1.75 (1.33, 2.32) |  | 1.63 (1.23, 2.17) |  | 1.37 (1.01, 1.85) |  |

Model 1: unadjusted analysis. Model 2: adjusted for age, sex, educational level and marital status. Model 3: adjusted for age, sex, educational level, marital status, hematoma volume, anatomical distribution, white matter lesions, encephalatrophy (linear measurements and visual templates). mRS, the modified Rankin Scale. OR, odds ratio. CI, confidence interval. *P*<0.05 indicates statistical significance.
